# Biomarkers in oral squamous cell carcinoma: A systematic review

**DOI:** 10.4317/medoral.27565

**Published:** 2025-08-16

**Authors:** Mónica López-Galindo, Margot de Sousa-Roques

**Affiliations:** 1 Associate Professor. PhD. Dentistry Department. Faculty of Health Sciences. Universidad Europea de Valencia, Spain; 2 Dentist graduated from Universidad Europea de Valencia, Spain

## Abstract

**Background:**

Oral squamous cell carcinoma (OSCC) is one of the most aggressive cancers in the oral cavity, often diagnosed at advanced stages, leading to significantly reduced survival rates. Traditional diagnostic methods, such as clinical examination and histopathology, have limitations in detecting early stages and assessing tumor variability. Molecular biomarkers, however, have shown great potential in overcoming these limitations by improving early diagnosis, prognosis, and personalized treatment. These biomarkers, when integrated with the TNM staging system, may provide more accurate and personalized clinical management.

**Material and Methods:**

A systematic review was conducted by searching PubMed, Scopus, and Web of Science databases for studies on biomarkers in OSCC published between January 2018 and December 2024. The studies were selected based on strict inclusion and exclusion criteria, focusing on those that investigated biomarkers related to OSCC diagnosis, prognosis, and therapeutic implications.

**Results:**

This review includes 10 studies involving 1024 patients with OSCC. Key biomarkers such as Ki67, HSP60, Survivin, E-cadherin, and PD-L1 were significantly associated with tumor progression, lymph node metastasis, and poor prognosis. The combined use of these biomarkers with traditional histopathological methods could enhance diagnostic accuracy, allowing for better patient stratification and more targeted treatment approaches. Additionally, saliva-based biomarkers have emerged as a promising, non-invasive diagnostic tool with high sensitivity and specificity for early detection of OSCC.

**Conclusions:**

The identification of specific biomarkers can significantly enrich the diagnostic, prognostic, and therapeutic management of OSCC, complementing the TNM staging system. These biomarkers are linked to critical clinical variables such as metastasis, survival, and response to treatment. Saliva-based biomarkers hold promise due to their non-invasive nature, but further validation through multicenter studies and standardization is required for their widespread clinical adoption.

## Introduction

Oral squamous cell carcinoma (OSCC) represents about 90% of oral cancers and remains one of the most common cancers in the head and neck region. Despite advances in treatment, the 5-year survival rate remains low, mainly due to the late detection of the disease (1). Early detection is crucial as it can significantly improve survival rates, particularly when diagnosed at an early stage, where survival rates can exceed 85% (2). However, OSCC is often diagnosed at an advanced stage, limiting therapeutic options and negatively impacting clinical outcomes (3).

Molecular biomarkers have emerged as a promising solution to improve early diagnosis, prognosis, and management of OSCC (4 - 6). A suggestive aspect of biomarker use is their detection in non-invasive body fluids like saliva. Studies have demonstrated that salivary biomarkers, such as IL-8 and MMP-1 proteins, are frequently associated with OSCC and can differentiate OSCC patients from healthy individuals . While salivary biomarkers show great potential for early detection, further research is needed to validate their use in large-scale (7) clinical trials (5).

Integrating biomarkers into the current TNM staging system could improve diagnostic accuracy by providing insights into the molecular biology of the tumor, such as depth of invasion (DOI) and extracapsular spread of lymph nodes, which are not always well accounted for in the current classification (8). Although biomarkers offer a new avenue for better clinical management of OSCC patients, their implementation in clinical practice requires additional studies and validation of these biomarkers in larger cohorts (2 , 7).

## Material and Methods

The present systematic review was carried out following the statement of the PRISMA Guide (Preferred Reporting Items for Systematic reviews and MetaAnalyses) (9)

Protocol and focus question

The Medline-PubMed database (United States National Library of Medicine) were used to search for indexed articles that evaluated biomarkers in patients with oral squamous cell carcinoma, published from January 2018 to December 2024, to answer the following question: In patients with oral squamous cell carcinoma, does the identification of specific biomarkers, compared to conventional clinical and histopathological methods, improve diagnosis, prognosis, and therapeutic management?

This study question was set according to the PICO structured question. The question format was established as follows:

P: Patients with oral squamous cell carcinoma

I: Identification of specific biomarkers for diagnosis

C: Conventional clinical and histopathological methods with the TNM classification (without biomarkers)

O: Improvement in diagnosis and prognosis

Selection criteria

Inclusion criteria:

Type of study: Publications in English, cohort studies, cross-sectional, and case-control studies.

Type of Population: Patients with oral squamous cell carcinoma (OSCC), diagnosed with clinical and histopathological methods, and those evaluated using molecular biomarkers.

Type of intervention: Assessing specific biomarkers in oral squamous cell carcinoma, comparing conventional diagnostic methods (clinical and histopathological) with biomarker analysis, focusing on salivary biomarkers for early diagnosis and prognosis.

Type of results: Articles evaluating the concentration levels of different salivary biomarkers/proteins in patients with oral squamous cell carcinoma, including comparisons between advanced and early stages, and assessing their correlation with clinical parameters (e.g., TNM classification).

Exclusion criteria:

Exclusion criteria were as follows: Systematic reviews, case reports, expert opinions, unpublished articles, in vitro studies, animal studies, studies not directly related to oral squamous cell carcinoma (OSCC), studies not using the updated TNM classification (2018), and studies with unclear or unvalidated methodologies.

Search strategy

An electronic search was conducted in Medline-PubMed database with the aim of identifying studies related to biomarkers and oral squamous cell carcinoma (OSCC). The search strategy was guided by the PICO question. The search terms used included: "oral squamous cell carcinoma," "biomarker," "protein," "salivary biomarkers," "saliva," "unstimulated saliva," "stimulated saliva," "early diagnosis," "prognosis," "TNM classification," "cancer markers," "molecular markers," "oral cancer," and "diagnostic biomarkers." These terms were combined using the operators "AND" and "OR." Filters applied included articles published between 2018 and 2024, in English.

Selection process

The study selection process was carried out in two stages. First, duplicates were removed using Mendeley, followed by the initial stage of screening. The title and abstract of each article were reviewed, and the inclusion and exclusion criteria were applied. In the second stage of screening, the full text of each article was read, and the inclusion and exclusion criteria were re-applied. Articles that did not meet the inclusion criteria were discarded and documented in a table that included the author's name, publication year, article title, and the reason for exclusion. The remaining articles that met the inclusion criteria were included in the systematic review. Each step was documented in the PRISMA 2020 flow diagram.

Data collection

The selection of the studies was carried out by two reviewers (ML, MDS). A selection process was followed to identify relevant studies. The following data were extracted from the included studies: author's surname and year of publication, age of subjects, gender, subjects with oral squamous cell carcinoma (OSCC), cancer staging (TNM classification). This data extraction was crucial for evaluating the relevance of the biomarkers and their association with OSCC diagnosis, prognosis and management.

Risk of bias tool and quality assessment

To measure the quality of non-randomized observational studies, the Newcastle-Ottawa Scale was used; "low risk of bias" was considered in the case of a 7-9, "intermediate" for 5-6 points and "high risk of bias" in the case of of 4. The Newcastle-Ottawa Scale was used to assess the quality of the included studies (Table 1 and Table 2).

Table 1 Table 2

Data synthesis

The extracted data were divided into qualitative and quantitative variables. The qualitative variables included the following: author's surname and year of publication, gender, subjects with oral squamous cell carcinoma (OSCC), cancer staging (TNM classification) and methods used to assess the biomarkers. The quantitative variables included sample size (number of participants), age and gender. Additionally, we extracted information on the biomarkers assessed, including the type of protein or biomarker identified, and the methods used to evaluate these biomarkers.

## Results

Biomarkers for diagnosis and prognosis in OSCC

This systematic review included 10 relevant studies that focused on various biomarkers associated with oral squamous cell carcinoma (OSCC), including Ki67, HSP60, Survivin, PD-L1, E-cadherin, Cathepsin B and Stathmin (Table 3).

Table 3

Ki67 and prognosis in OSCC

The Ki67 labeling index (LI) was significantly higher in OSCC tissues compared to normal tissues. Ki67, a marker of cellular proliferation, was associated with tumor aggressiveness and poor prognosis. The study by Gadbail et al. (2021) reported that Ki67 expression was elevated in advanced stages of OSCC and was correlated with nodal metastasis and reduced survival rates. Additionally, Ki67 was shown to be a reliable biomarker for assessing the proliferative potential of OSCC cells and their correlation with poor clinical outcomes (10).

HSP60 and survivin expression

HSP60 and Survivin were frequently overexpressed in OSCC tissues, particularly in high-grade tumors. Overexpression of HSP60 was linked to tumor progression and metastasis. The study by Vadla et al. (2022) showed that high HSP60 expression was correlated with poor survival outcomes, indicating its role in the aggressive behavior of OSCC (11). Similarly, Survivin expression was significantly higher in OSCC tissues and associated with advanced disease stages and poor prognosis (12).

PD-L1 and PD-1 in OSCC

The expression of PD-L1 and PD-1 was significantly elevated in OSCC tissues. PD-L1 was overexpressed in 64.9% of OSCC cases, while PD-1 was similarly elevated in 61.9% of patients. These markers were associated with cervical lymph node metastasis and poor prognosis. The findings by Maruse et al. (2018) demonstrated that high expression of PD-L1/PD-1 was linked to immune evasion and poor survival in OSCC patients (13).

E-cadherin downregulation

E-cadherin, a key cell adhesion molecule, was downregulated in OSCC tissues, particularly in tumors with metastatic potential. The loss of E-cadherin expression was associated with the epithelial-mesenchymal transition (EMT), a process involved in tumor invasion and metastasis. The study by López-Verdín et al. (2019) found that decreased E-cadherin expression correlated with poor differentiation and increased metastatic potential in OSCC (14).

Stathmin (Op18) and tumor progression

Stathmin (Op18) was found to be overexpressed in OSCC tissues, particularly in high-grade tumors and those with lymph node metastasis. Overexpression of Stathmin was linked to poor clinical outcomes, and its role as a prognostic biomarker for OSCC was highlighted (15).

Salivary biomarkers

Cathepsin B, a protease involved in tumor invasion and metastasis, was significantly elevated in the saliva of OSCC patients, particularly those with high tumor grade and lymph node metastasis. The study by Vadla et al. (2022) reported that Cathepsin B in saliva showed high sensitivity and specificity for diagnosing OSCC, suggesting its potential for early detection and monitoring of disease progression (16).

Statistical analysis

The statistical analysis showed significant correlations between Ki67, HSP60, and PD-L1 with poor prognosis. For instance, patients with high Ki67 LI had significantly lower 3-year survival rates (37.7%) compared to those with low Ki67 LI (96.2%) (10). Additionally, PD-L1 and Survivin overexpression were associated with shorter overall survival (OS) and disease-free survival (DFS) (12 , 13).

## Discussion

This review examined several biomarkers associated with oral squamous cell carcinoma (OSCC), highlighting their diagnostic, prognostic, and therapeutic roles. Biomarkers such as Ki67, HSP60, Survivin, PD-L1, E-cadherin, Stathmin, and Cathepsin B have shown significant associations with tumor progression, metastasis, and poor prognosis, supported by the results from the studies analyzed. However, several new biomarkers are also emerging as potential candidates, including ALPK1, DPP-4, and TGM-3, which bring new perspectives to the field of OSCC.

The proliferation marker Ki67 remains one of the most widely used biomarkers to predict the aggressiveness of OSCC. High expression of Ki67 is associated with advanced disease stages, lymph node metastasis, and reduced survival (4 , 5 , 10 , 13). This conclusion is confirmed by several studies analyzed in this review, which underline its central role in determining the prognosis of OSCC patients. Ki67 is now widely used to assess cellular proliferation and the risks of tumor progression, and its measurement could be used to personalize therapeutic strategies (3 , 6 , 17).

HSP60 and Survivin are significant biomarkers in OSCC, mainly due to their association with tumor progression and resistance to treatment. HSP60, as a stress response protein, is involved in the survival of tumor cells under stress and contributes to cancer progression (4 , 5 , 11 , 18). The overexpression of Survivin is strongly linked to resistance to apoptosis and poor response to conventional treatments, making it a potential therapeutic target to improve treatment outcomes in OSCC (12 , 19).

PD-L1 and its receptor PD-1 have been identified as key immune evasion mechanisms in OSCC. The results show that PD-L1 is overexpressed in a significant number of OSCC cases, and this overexpression is linked to advanced stages and poor survival (3 - 6 , 13 , 20). This signaling pathway is thus central to the development of immunological therapies, as demonstrated by recent work on immune checkpoint inhibitors, which show potential improvement in outcomes for patients with OSCC who have failed conventional therapies (21).

The loss of E-cadherin expression, a marker of cell adhesion, remains one of the key mechanisms underlying epithelial-to-mesenchymal transition (EMT), a process involved in the migration and metastasis of tumor cells. In the studies we examined, the loss of E-cadherin has been consistently associated with advanced OSCC stages and faster tumor progression (4 , 5 , 14 , 22). This observation is reinforced by recent research showing that the restoration of E-cadherin could inhibit tumor migration and provide a potential therapeutic approach to slow OSCC progression (23).

Stathmin (Op18) is another interesting biomarker involved in regulating microtubule dynamics and cellular proliferation. High Stathmin expression has been associated with advanced-stage OSCC, particularly in patients with lymph node metastasis (15 , 24). This protein has also shown a correlation with poor survival, suggesting it could be used as a prognostic biomarker in OSCC (23 , 25). The growing interest in Stathmin as a biomarker for treatment resistance opens new therapeutic avenues, particularly in targeted therapies.

Salivary biomarkers are particularly promising for the early diagnosis of OSCC, and biomarkers such as Cathepsin B have shown the ability to identify early stages of the disease. Analysis of Cathepsin B in saliva revealed a significant correlation with histopathological grade and the presence of lymph node metastasis, making it a potential biomarker for non-invasive OSCC monitoring (16 , 26). The results of Jain et al. (2021) show that these salivary biomarkers are particularly sensitive for early detection, offering a more accessible diagnostic tool for patients (27).

Emerging biomarkers such as ALPK1, DPP-4, and TGM-3 offer new perspectives for OSCC management. ALPK1 has shown a significant association with lymph node metastasis and poor prognosis in OSCC tumors (20 , 28). Similarly, DPP-4, measured in serum and saliva, has proven its ability to differentiate between precancerous and cancerous lesions, which could revolutionize OSCC diagnosis (29). Finally, TGM-3, expressed in tumor tissues and serum, has shown strong prognostic value in the management of OSCC patients undergoing chemotherapy and radiotherapy (18 , 30).

Despite the enormous potential of these biomarkers, several challenges remain. Variability in biomarker detection methods, particularly for salivary biomarkers, constitutes a major limitation for their clinical use. Standardizing measurement techniques and analysis protocols is essential to ensure the reliability and reproducibility of results. Furthermore, the absence of highly specific biomarkers for OSCC suggests that a combined panel may improve diagnostic accuracy. Future large-scale clinical studies, involving diverse patient cohorts, are needed to validate these biomarkers and their ability to predict treatment response and monitor disease progression (29 , 31 - 34).

## Conclusions

This study highlights the significance of biomarkers such as Ki67, HSP60, Survivin, Stathmin, E-cadherin, PD-L1, and Cathepsin B in OSCC. These biomarkers provide essential insights into tumor behavior and prognosis, offering the potential for improved diagnosis and personalized treatment strategies. Further research is needed to validate their clinical applicability and to explore their role in monitoring treatment responses and disease progression.

## Figures and Tables

**Table 1:** Table Measurement of the risk of bias in non-randomized observational studies using the Newcastle-Ottawa scale - cohort observational studies without a control group.

	Cohort representativeness	Selection of the unexposed cohort	Exposure verification	Demonstration of the absence of the variable of interest at the beginning. 4o mini	Comparability (most important factor)	Comparability (other factors)	Measurement of outcomes	Sufficient follow-up	Dropout rate	Total
Shiying Shen y cols 2024 (32)	X	-	X	X	X	X	X	X	-	7
Amol Ramchandra Gadbail y cols 2021 (10)	X	-	X	X	X	X	X	X	-	7

1

**Table 2:** Table Measurement of the risk of bias in non-randomized observational studies using the Newcastle-Ottawa scale - observational studies with a non-randomized control group.

	Definition of the cases	Representativeness	Selection of the controls	Definition of the controls	Comparability (most important factor)	Comparability (any other variable)	Verification of exposure	Same method for both groups	Dropout rate	Total
Ying Zhou y cols 2023 (11)	X	X	X	X	X	X	X	X	-	7
Cheng-Lin Wu y cols 2022 (33)	X	X	X	X	X	X	X	X	-	7
Alveena Shabbir y cols 2022 (33)	X	X	X	X	X	X	X	X	-	7
Ignacio GonzÃ¡lez Segura y cols 2022 (16)	X	X	X	X	X	X	X	X	-	7
Shigeru Sakurai y cols 2024 (34)	X	X	X	X	X	X	X	X	-	7
Purnima Vadla y cols 2022 (15)	X	X	X	X	X	X	X	X	-	7
Sandra LÃ³pez-VerdÃ­n y cols 2019 (14)	X	X	X	X	X	X	X	X	-	7
Y. Maruse y cols 2018 (13)	X	X	X	X	X	X	X	X	-	7

2

**Table 3:** Table Characteristics of the reviewed studies.

Article	N°	Biomarkers analyzed	Techniques used	Associated risk factors	Main findings	
Shiying Shen y cols (32)	338	CD274, HAVCR2, LAG3, TP53, CDKN2A, NOTCH1	Bioinformatics analysis	-	Anomalous expression of lactic acid metabolism genes in COCE (p<0.05)	
Ying Zhou y cols (11)	79	HSP60, Survivin	Bioinformatics analysis Immuno-histochemistry	Tobacco (60.8 %)Alcohol (51,9%)Areca nut (43%)	Increased expression of HSP60 in patients with metastasis and stage III-IV Survivin is highly expressed in poorly differentiated tumors. (p<0.05)	
Cheng-Lin Wu y cols (33)	21	CK13, CK17, Ki-67, Ln5Î³2	Bioinformatics analysisImmuno-histochemistry Scoring system	Areca nut	96% score >=9, indicating a high risk of malignancy (p<0.05)	
Alveena Shabbir y cols (12)	60	Cathepsin B	Sandwich ELISAStatistical analysis	TobaccoAreca nutGeneticsUV radiationNutritional deficiencies	Higher salivary cathepsin B levels in patients with OSCC Correlation between cathepsin B and histological grade (good>poor>moderate)Cathepsin B levels increase with tumor size Cathepsin B could be a useful non-invasive salivary biomarker for early diagnosis of OSCC.(p<0.001)	
Ignacio GonzÃ¡lez Segura y cols (16)	46	Connexin 43, Bcl-2, Bax, Ki67, E-cadherin	Immuno-histochemistryPCR-RFLPStatistical analysis	Tobacco Alcohol	Altered expression of cell adhesion biomarkers Cx43 and E-cadherin suggesting a loss of cell adhesion favoring tumor invasion.Bcl-2 and Ki67 overexpression indicating proliferation and inhibition of apoptosis, Bax confirming suppression of apoptosis. (p<0.05)	
Shigeru Sakurai y cols (34)	96	Integrins αV, β and FAK, pFAK	Immuno-histochemistry Statistical analysis	-	Invasion-related integrin expressionIntegrin β8 is associated with an increased risk of lymph node metastasis.pFAK (phosphorylated FAK) is associated with significantly reduced overall survival of patients. (p<0.05)	
Purnima Vadla y cols (15)	30	Stathmin (Op18)	Immuno-histochemistryHistopathological analysisStatistical analysis	-	Stathmin associated with tumor progression and TNM stagingCorrelation between Stathmin expression and tumor grade (well<moderate<poor) Stathmin expression is higher in poorly differentiated SCC and when metastases are present. Stathmin increases in advanced stages of ESCC (III-IV)	
Sandra LÃ³pez-VerdÃ­n y cols (14)	40	E-cadherin (CDH1)	qRT-PCRImmuno-histochemistry Statistical analysis	Tobacco Alcohol	Reduced E-cadherin expression in ESCC.Patients with metastases have lower E-cadherin expression.Weak positive correlation between E-cadherin expression and tumor size (E-cadherin T).weak negative correlation (rho=-0.389, p=0.001) between stage and E-cadherin expression (Estadio E-cadherin) (p<0.05)	
Y. Maruse y cols (13)	97	PD-L1, PD-1, CD25	Immuno-histochemistry Statistical analysis	-	Elevated PD-L1/PD-1 expression correlated with lymph node metastasis (PD-1, located on T lymphocytes, is activated by PD-L1, reducing its ability to attack the tumor).Group C (OR=3.99, p=0.035): >1 there is association and 3.5% that it is due to chance. (p<0.05)	
Amol Ramchandra Gadbail y cols (10)	217	Ki67	Immuno-histochemistry Statistical analysis	TobaccoBetel nut	Ki67 expression when tumor differentiation (poor>moderate>good)Ki67 expression higher in advanced stages (III-IV) than in early stages (I-II).Significant correlation between Ki67 expression and tumor progression (p<0.001).Elevated Ki67 expression is associated with an increased risk of lymph node metastasis.	

3

## Data Availability

Declared none.
